# Supplementary material for: A Refined Method for Studying Foraging Behaviour and Body Mass in Group-Housed European Starlings
Source: Animals (Basel). 2022 Apr 29;12(9):1159. doi: 10.3390/ani12091159 (PMC9099603; doi:10.3390/ani12091159)
Supplement: Supplementary file 1 [file animals-12-01159-s001.zip › Supplementary files/Supplementary Figures.pdf]

Supplementary Figures S1-S11

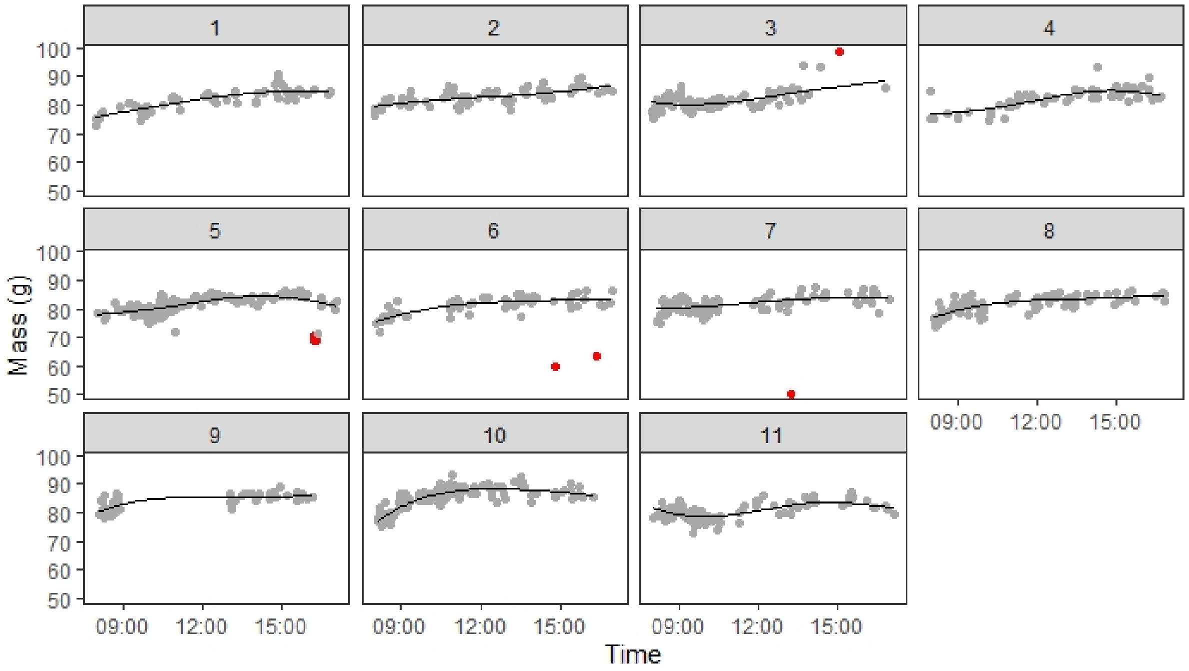

Figure S1. Raw mass data from bird P74. Panels 1-11 show scatterplots of individual stable mass measurements (g) by time of day (hours) for 11 consecutive days of continuous foraging under closed economy. Red points indicate masses that were excluded from the final model due to falling more than 10 g from the initial cubic fits (see Methods for details). The black line shows the cubic fit to the remaining grey points that was used to derive estimated masses for specific times of day.

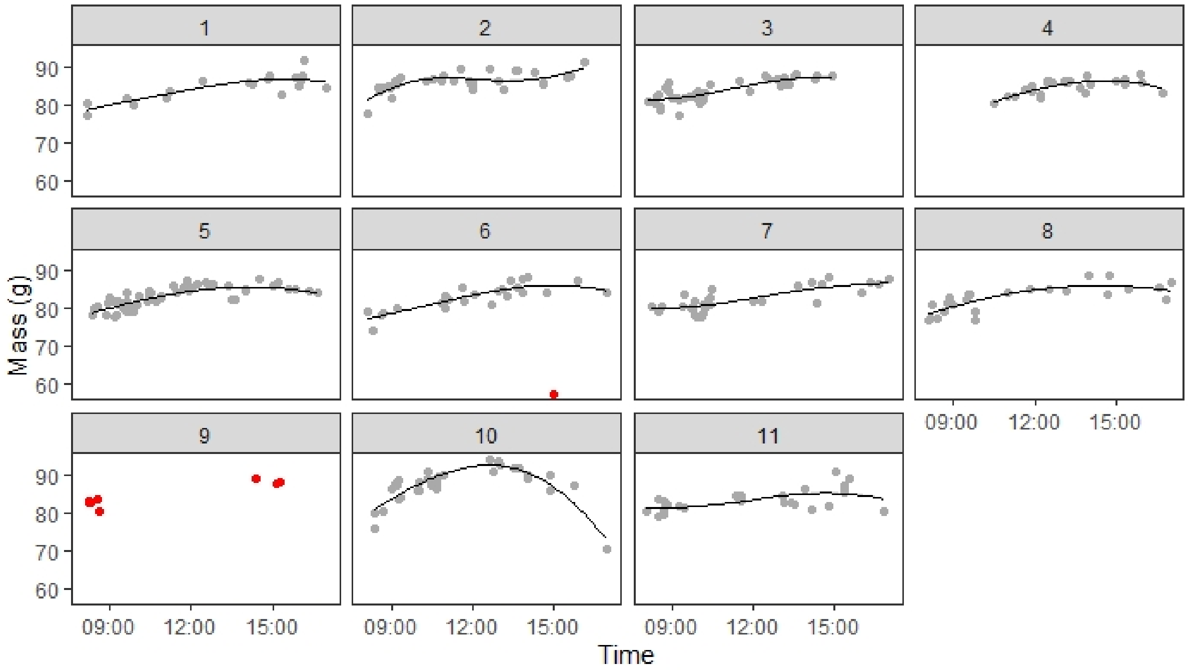

Figure S2. Raw mass data from bird P75. For details see Figure S1.

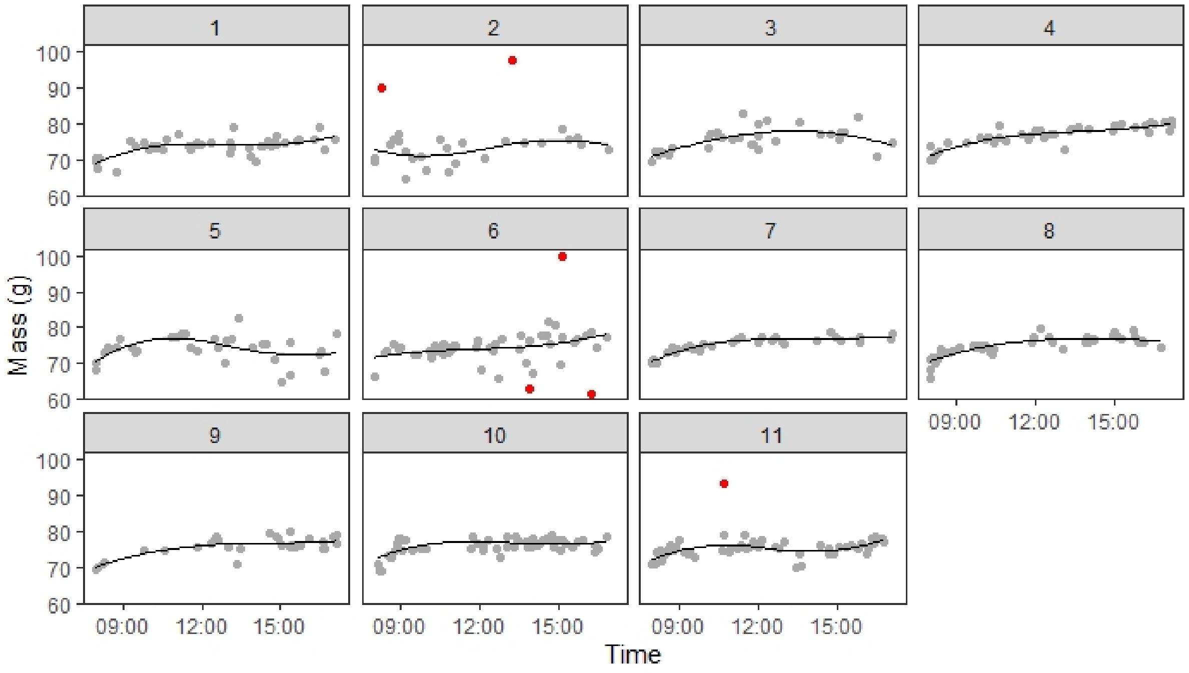

Figure S3. Raw mass data from bird P78. For details see Figure S1.

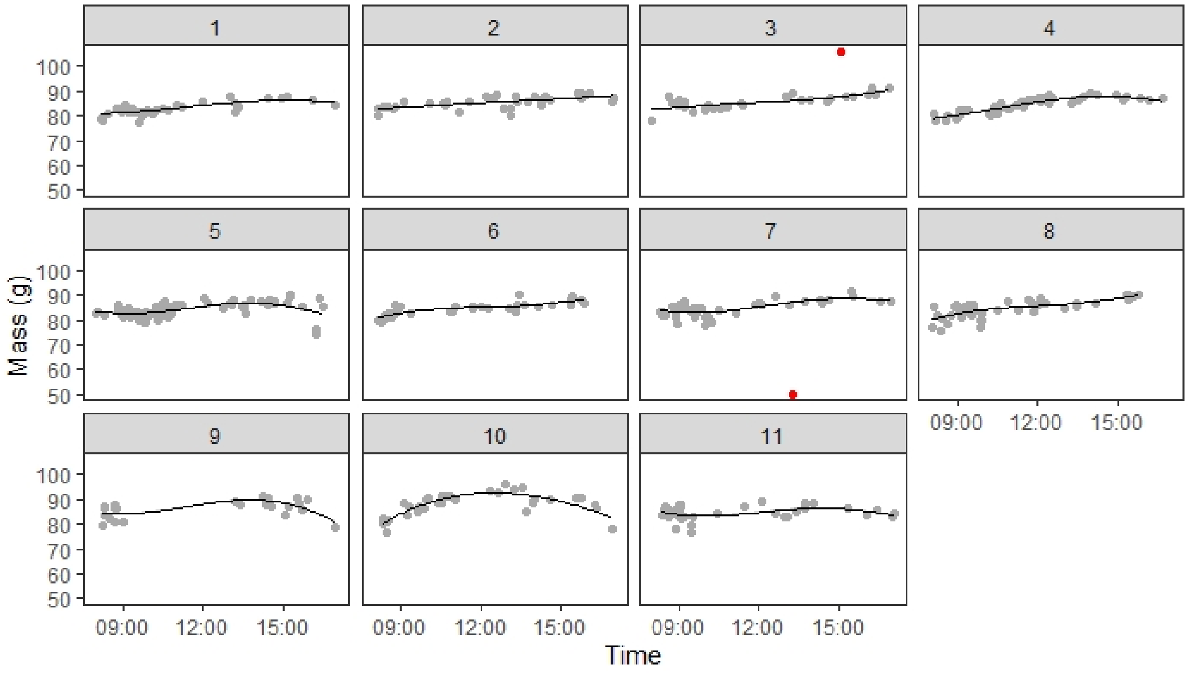

Figure S4. Raw mass data from bird P79. For details see Figure S1.

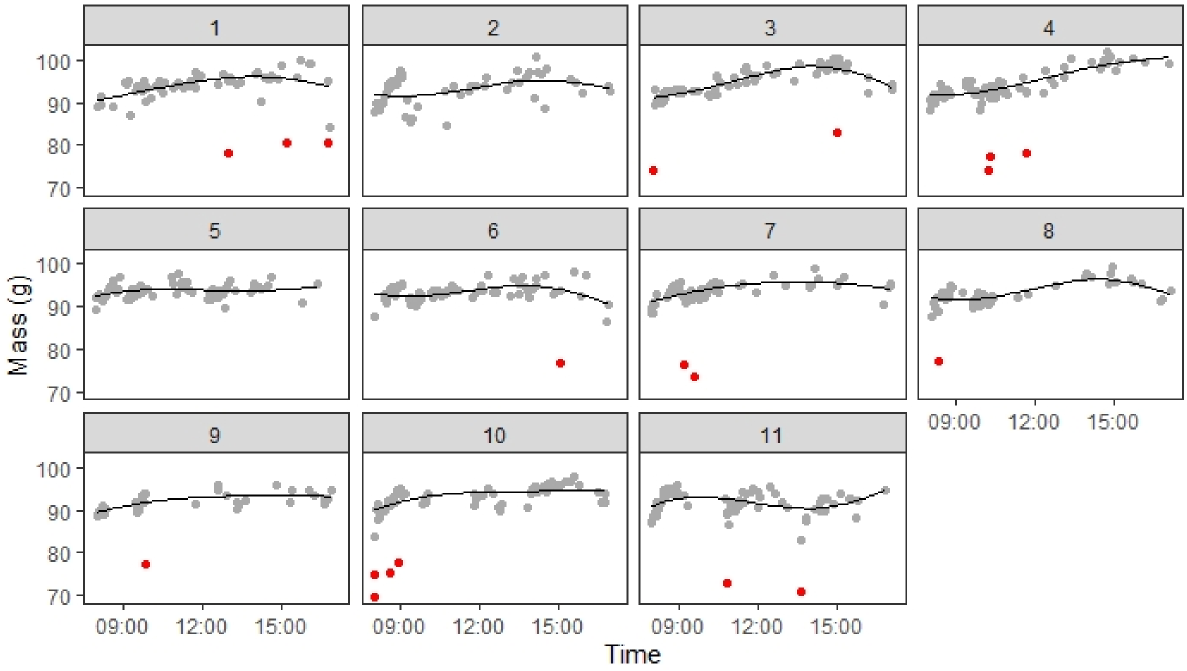

Figure S5. Raw mass data from bird P80. For details see Figure S1.

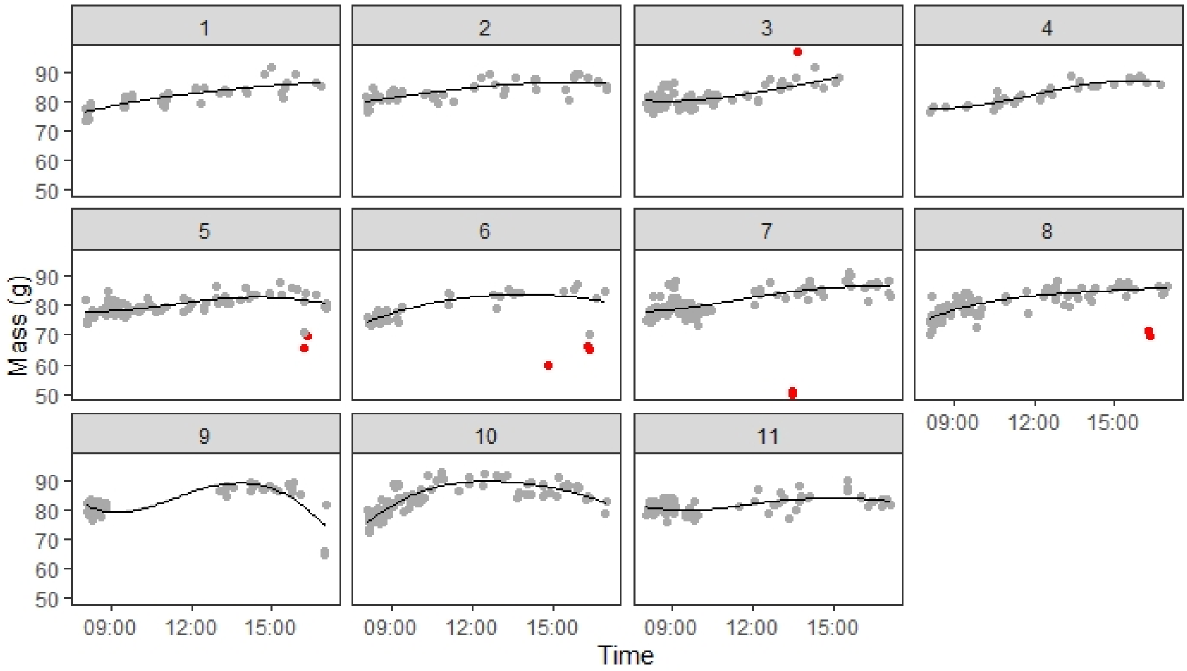

Figure S6. Raw mass data from bird P91. For details see Figure S1.

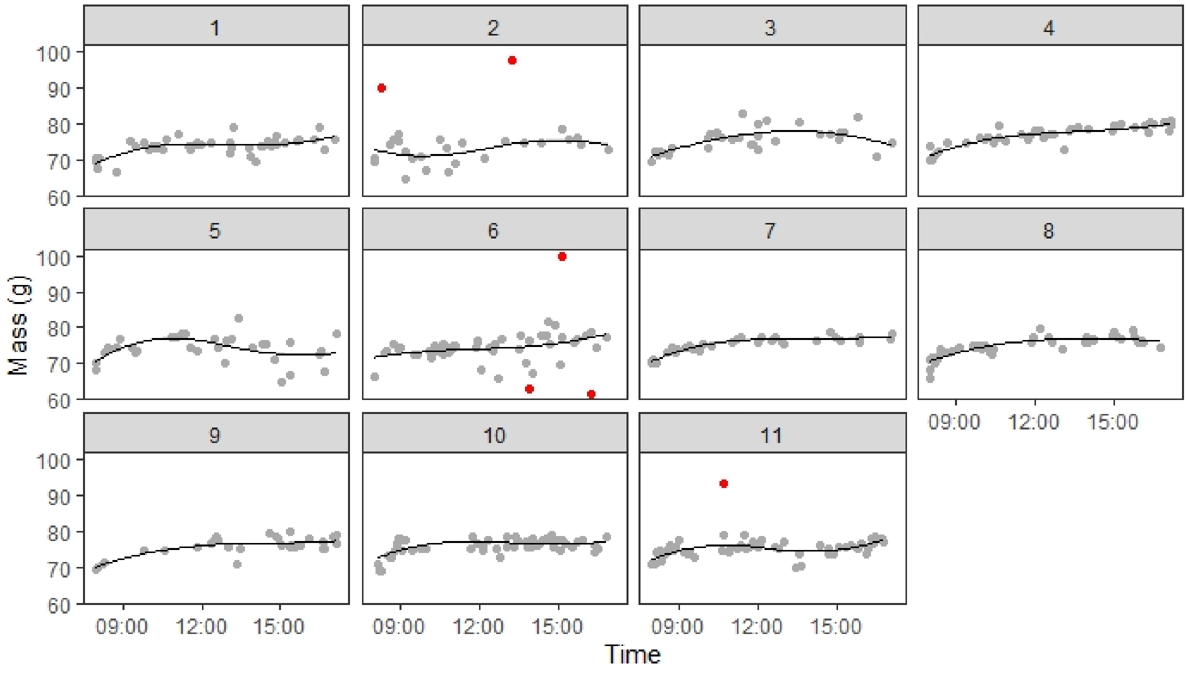

Figure S7. Raw mass data from bird P92. For details see Figure S1.

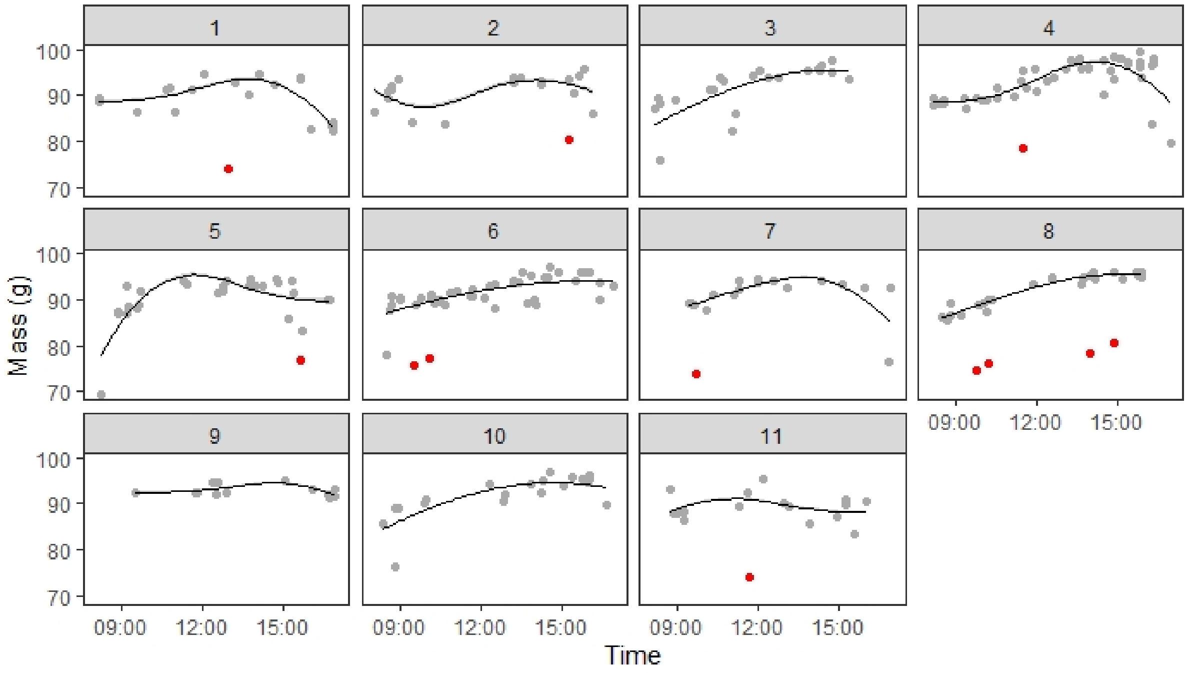

Figure S8. Raw mass data from bird P93. For details see Figure S1.

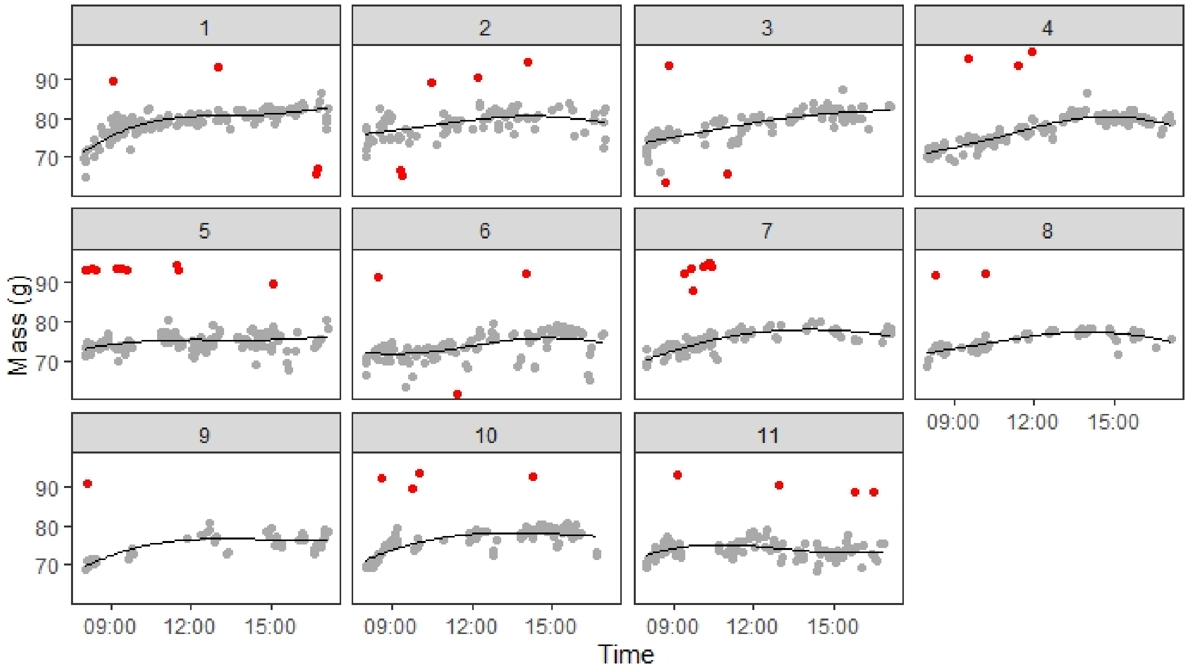

Figure S9. Raw mass data from bird P95. For details see Figure S1.

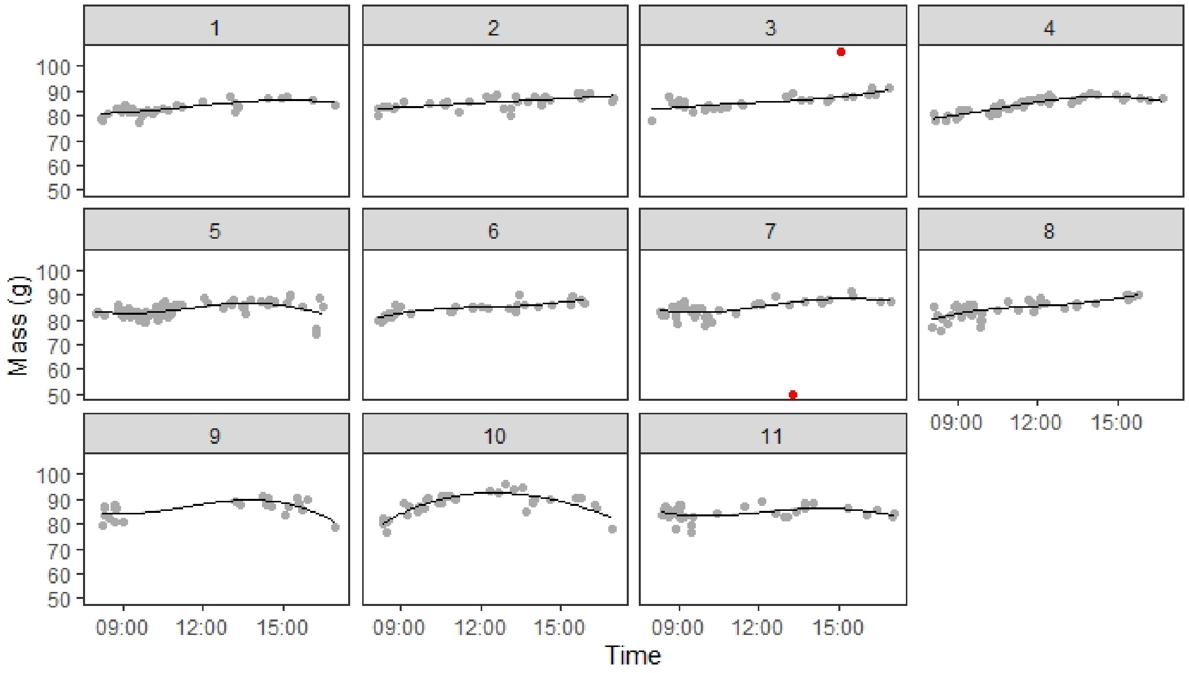

Figure S10. Raw mass data from bird P98. For details see Figure S1.

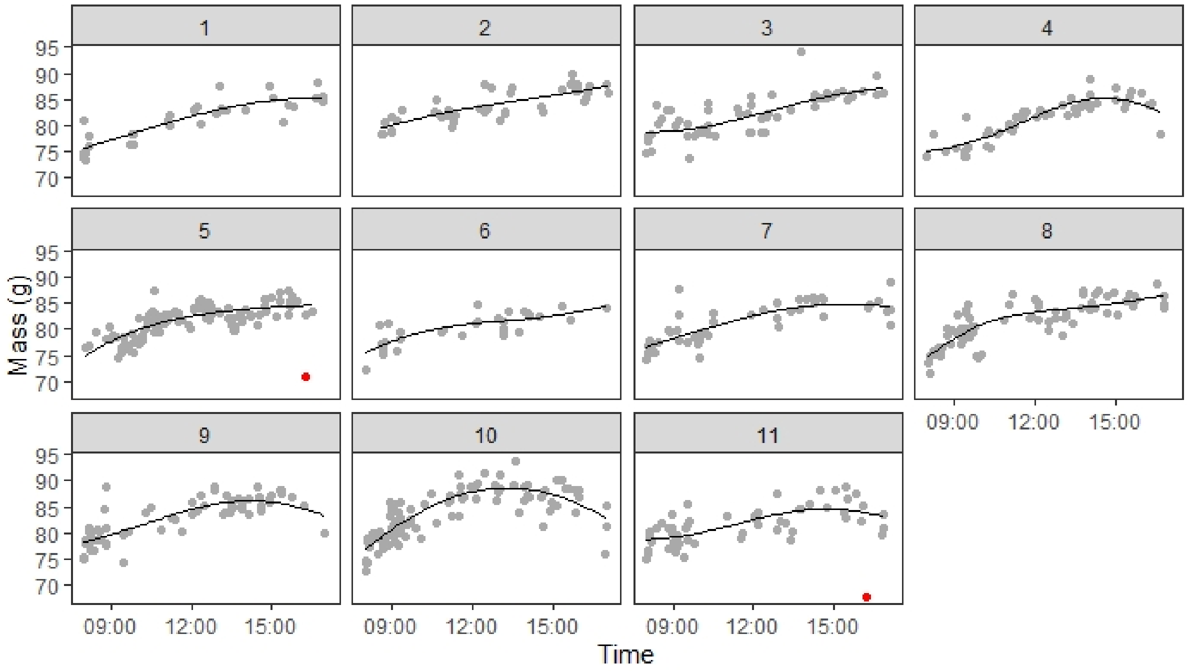

Figure S11. Raw mass data from bird P99. For details see Figure S1.
