# Supplementary material for: A Refined Method for Studying Foraging Behaviour and Body Mass in Group-Housed European Starlings
Source: Animals (Basel). 2022 Apr 29;12(9):1159. doi: 10.3390/ani12091159 (PMC9099603; doi:10.3390/ani12091159)
Supplement: Supplementary file 1 [file animals-12-01159-s001.zip › Supplementary files/Welfare monitoring in European starlings.pdf]

Experimental birds in aviaries equipped with the Social Foraging System (SFS)

1. Welfare monitoring arrangements for birds not yet visiting the SFS by researchers responsible

A. At least twice daily, am (0900-1100) and pm (1200-1400): visually inspect birds and complete CBC room books.

B. Daily: calculate total daily crumb consumption per bird in the room and record on daily welfare score sheet (may be for previous day depending on when food is weighed).

C. At least every 2 days, pm: catch and manually weigh any birds that have not started visiting SFS. Record manual masses on daily welfare score sheet.

D. At start and end of experiment: manually weigh birds and record masses on daily welfare score sheet.

- **Observation**: Bird has puffed feathers and is lethargic or struggles to fly/get off the ground.
  - **Actions**: Immediately catch bird and manually weigh. Remove bird from experimental protocol and give *ad lib*. food including mealworms in bowls immediately for the rest of the day. Observe whether bird eats. Inform Licence Holder/Deputy, NACWO and NVS.
- **Observation**: Bird(s) have not eaten any crumb in the previous 24 hours (despite having *ad libitum* food available via the SFS). Note that this situation often arises when birds are first moved to experimental aviaries/crumb diet and is more common in birds that are heavy (>80g) going into experiments.
  - **Actions**: Visually inspect bird(s) carefully for signs of poor welfare. Check most recent weight records. If a bird has not been weighed within the past 2 days, or it has been weighed and this weight was less than 80g, then catch, weigh and, if necessary, go to actions following low weights detailed below. If the bird has been weighed within the past 2 days and its weight was above 80g then no further action.
- **Observation**: Bird is less than 65g, but otherwise healthy looking. NB this bird is light and needs close attention.
  - **Actions**: Check historical weight data on bird to determine whether it is abnormally light. If the weight is abnormally low, or if in any doubt, stop experimental protocol and immediately give *ad lib*. food in bowls including mealworms for the rest of the day. Inform Licence Holder/Deputy to discuss further action.
- **Observation**: Bird is less than 62g, but otherwise healthy looking. NB this bird is very light and immediate action is necessary.
  - **Actions**: Stop experimental protocol and immediately give *ad lib*. food in bowls including mealworms for the rest of the day. Observe whether bird eats when first given bowls. Inform Licence Holder/Deputy to discuss further action.

2. Welfare monitoring arrangements for birds foraging from the SFS by researchers responsible

A. At least twice daily, am (0900-1100) and pm (1200-1400): visually inspect birds and complete CBC room book(s).

B. Daily: calculate total daily crumb consumption per bird in the room and record on daily welfare score sheet (may be for previous day depending on when food is weighed).

C. Daily, pm (1200-1400): check whether all birds have been observed visiting the SFS that day; calculate and record number of mass events and mean of last 5 observed masses for each bird on daily welfare score sheet.

D. At end of experiment: manually weigh birds and record masses on daily welfare score sheet.

- **Observation**: Bird has puffed feathers and is lethargic or struggles to fly/get off the ground.
    - **Actions**: Immediately catch bird, inspect and manually weigh to double-check weight. Remove bird from experimental protocol immediately and give *ad lib*. food in bowls including mealworms for the rest of the day. Observe whether bird eats from bowls. Inform Licence Holder/Deputy, NACWO and NVS.
- **Observation**: Bird has not been detected by the SFS that day at pm check, but otherwise healthy looking.
    - **Actions**: Visually inspect bird again for signs of poor welfare and try to observe whether rings with RFID tags are still present (they can fall off). Catch bird, check both tags are working with manual scanner and manually weigh. Replace RFID tag(s) that are missing/not working. If weight is OK then no further action. If weight is below 65g then see actions below.
- **Observation**: Mean of last 5 masses at pm check (or manual weight) is less than 65g, but otherwise healthy looking. NB this bird is potentially light and needs close attention that day to establish whether there is a problem.
    - **Actions**: Establish whether all the birds in the room are lighter than normal or just one. If all birds are light then this suggests an equipment problem (feeder not working or inaccurate balance). Double-check all equipment and test/calibrate SFS balances. If in any doubt about accuracy of balances catch and manually weigh all birds. If just one bird is light check historical weight data for bird to see whether weight is abnormally low. Visually inspect bird again for any signs of poor welfare. Inform Licence Holder/Deputy to discuss further action.
- **Observation**: Mean of last 5 masses at pm check (or manual weight) is less than 62g, but otherwise healthy looking. NB this bird is potentially very light and immediate action is necessary.
    - **Actions**: Establish whether all the birds in the room are lighter than normal or just one. If all birds are light then this suggests an equipment problem (feeder not working or inaccurate balance). Double-check all equipment and test/calibrate SFS balances. If in any doubt about accuracy of balances catch and manually weigh all birds. If low weight is confirmed, stop experimental protocol and immediately give *ad lib*. food in bowls including mealworms for the rest of the day. Observe whether light bird eats when first given bowls. Inform Licence Holder/Deputy to discuss further action.

Daily welfare score sheet for starlings in SFS experiments

*Only required if specified in monitoring arrangements.

**Date:**			**Researcher responsible:**					
	**Bird**			**Mass data for birds visiting SFS only (pm check: 1200-1400 hrs)**				
**Room no.**	**Ring ID**	**RFID ring colour**	**Visiting SFS yet (Y/N)**	**Time of check**	**No. mass events**	**Mean of last 5 masses**	**Manual mass***	**Crumb eaten/bird in room**

Notes on bird welfare and any actions taken:
